# Supplementary material for: Beliefs of UK Transplant Recipients about Living Kidney Donation and Transplantation: Findings from a Multicentre Questionnaire-Based Case–Control Study
Source: J Clin Med. 2019 Dec 21;9(1):31. doi: 10.3390/jcm9010031 (PMC7019237; doi:10.3390/jcm9010031)
Supplement: Supplementary file 1 [file jcm-09-00031-s001.pdf]

Supplementary material

Supplementary Methods

The study was based at 14 UK hospitals: Belfast, Birmingham, Bristol, Cambridge, Epsom and St Helier, Guy's and St Thomas's, Imperial, Leicester, Manchester, Newcastle upon Tyne, Nottingham, Oxford, Sheffield, and St George's.

Table S1. Responders and non-responders

	**Total invited** n=3103 (%)	**Participants** n=1240 (%)	**Non-participants** n=1863 (%)[a]
**Sex**			
Male	1902 (61)	705 (57)	1197 (64)
Female	1201 (39)	514 (41)	687 (37)
Missing	0	21 (2)	0
**Renal Transplant type**			
Live-donor	1462 (47)	672 (54)	790 (42)
Deceased-donor	1641 (53)	565 (46)	1076 (58)
Missing	0	3 (0.2)	0

[a] As % were rounded up to the nearest whole number some totals are more than 100%. We could not analyse response by age as people were sampled by age at transplant and then asked to enter their current age in the questionnaire.

Table S2. Responders compared to national denominator population

**Characteristics**		**Study DDKT recipients** %	**National population of DDKT recipients 2013-2017** %	**Chi2 p-value**	**Study LDKT recipients** %	**National population of LDKT recipients 2013-2017** %	**Chi² p-value**
**Sex (%)**	**Male**<br>**Female**	58<br>42	62<br>38	0.56	58<br>42	59<br>41	0.89
**Age (years)**	**50-59**	28	26	0.75	27	23	0.51
	**60-69**	24	22	0.74	25	15	0.08
**Ethnicity (%)**	**White**<br>**BAME/Other**	82<br>18	73<br>27	0.13	89<br>11	83<br>17	0.22

Individuals with missing data for each characteristic excluded from figures to allow direct comparison to published NHS Blood and Transplant data

Supplementary Missing data

Case-control variable

0.24% (n=3) were missing a data on case-control status (transplant type: i) living-donor or ii) deceased-donor transplant). This was as a result of the questionnaire code not being entered by participants who chose to complete the questionnaire online. Participants for whom case-control status was not recorded were more likely to also have missing data for age (2/3 had age missing, Chi^2 p<0.001), sex (2/3 had sex missing, Chi^2 p<0.001), ethnicity (2/3 were missing ethnicity, Chi^2 p<0.001), and IMD rank quintile (2/3 were missing education, Chi^2 p<0.001).

Education

6.69% (n=83) missing overall.

Those with missing data for age, sex, ethnicity, and transplant type were more likely to have missing data for this variable. When focusing on individuals with recorded demographic data (age, sex, ethnicity and transplant type), no pattern of missingness with age (Chi^2 p=0.47), sex (Chi^2 p=0.12), or transplant type (Chi^2 p=0.17) but maybe with ethnicity (Chi^2 p<0.001) – suggested that black patients' and those from 'other ethnic groups' are more likely to have missing data.

Age

2.74% (n=34) missing overall.

Those with missing data for sex, ethnicity, education and transplant type were more likely to have missing data for this variable. When focusing on individuals with recorded demographic data, no pattern of missingness with sex (Chi^2 p=0.05), education (no participants with age missing but education recorded), ethnicity (Chi^2 p=0.97) or transplant type (Chi^2 p=0.89).

Sex

1.69% (n=21) missing overall.

Those with missing data for age, ethnicity, education and transplant type were more likely to have missing data for this variable. When focusing on individuals with recorded demographic data, no pattern of missingness with age, education, ethnicity or transplant type (Chi^2 p=0.75).

Ethnicity

2.42% (n=30) missing overall.

Those with missing data for age, ethnicity, education and transplant type were more likely to have missing data for this variable. When focusing on individuals with recorded demographic data, no

pattern of missingness with age (Chi^2 p=0.73), or transplant type (Chi^2 p=0.94), but maybe with sex (Chi^2 p=0.03) – women more likely to have missing data, but numbers very small. Only one person had missing ethnicity and education data.

Table S3a. Difference in beliefs with participant sex

Belief statement	Sex	Disagree (%)	Agree (%)	Don't know (%)	Chi^2 p-value
**1. It is morally acceptable to take a kidney from a healthy person.**	Female<br>Male	35 (7.0)<br>30 (4.3)	433 (86.6)<br>618 (88.7)	32 (6.4)<br>49 (7.0)	0.12
**2. Donors often agree to donate due to feelings of guilt or family pressure.**	Female<br>Male	251 (49.8)<br>378 (54.3)	108 (21.4)<br>120 (17.2)	145 (28.8)<br>198 (28.5)	0.15
**3. Donating a kidney is a rewarding experience for the live donors.**	Female<br>Male	14 (2.8)<br>12 (1.7)	405 (80.4)<br>591 (84.6)	85 (16.9)<br>96 (13.7)	0.13
**4. Donating a kidney to someone requires an extremely close personal relationship.**	Female<br>Male	340 (67.3)<br>471 (67.4)	125 (24.8)<br>168 (24.0)	40 (7.9)<br>60 (8.6)	0.90
**5. A living donor kidney transplant may strengthen the relationship between the donor and recipient.**	Female<br>Male	64 (12.7)<br>77 (11.0)	337 (66.7)<br>500 (71.5)	104 (20.6)<br>122 (17.5)	0.20
**6. Approaching a potential donor who then says no will change the relationship between the two people.**	Female<br>Male	221 (44.0)<br>336 (48.1)	100 (19.9)<br>125 (17.9)	181 (36.1)<br>238 (34.1)	0.37
**7. Asking someone to donate makes the recipient seem selfish.**	Female<br>Male	235 (46.7)<br>337 (48.4)	148 (29.4)<br>193 (27.7)	120 (23.9)<br>167 (24.0)	0.79
**8. It is acceptable for a parent to receive a kidney from his/her child (over 18 years old).**	Female<br>Male	69 (13.7)<br>54 (7.7)	361 (71.5)<br>567 (81.1)	75 (14.9)<br>78 (11.2)	<0.001
**9. Decisions about donation should be made by the donor alone. The recipient should not ask for a kidney.**	Female<br>Male	115 (22.8)<br>179 (25.6)	326 (64.7)<br>432 (61.9)	63 (12.5)<br>87 (12.5)	0.52
**10. Since the donor operation is not risk free, someone who needs a kidney transplant should wait for a kidney from someone who has died.**	Female<br>Male	420 (83.3)<br>575 (82.3)	34 (6.8)<br>41 (5.9)	50 (9.9)<br>83 (11.9)	0.49

Table S3b. Difference in beliefs with participant age

Belief statement	Age	Disagree (%)	Agree (%)	Don't know (%)	Chi^2 p-value
1. It is morally acceptable to take a kidney from a healthy person.	<60 years<br>≥60 years	46 (6.2)<br>18 (4.0)	643 (87.3)<br>397 (88.8)	48 (6.5)<br>32 (7.2)	0.25
2. Donors often agree to donate due to feelings of guilt or family pressure.	<60 years<br>≥60 years	420 (56.8)<br>202 (45.1)	143 (19.4)<br>83 (18.5)	176 (23.8)<br>163 (36.4)	<0.001
3. Donating a kidney is a rewarding experience for the live donors.	<60 years<br>≥60 years	20 (2.7)<br>6 (1.3)	612 (82.6)<br>375 (83.5)	109 (14.7)<br>68 (15.1)	0.30
4. Donating a kidney to someone requires an extremely close personal relationship.	<60 years<br>≥60 years	492 (67.4)<br>254 (70.8)	176 (24.1)<br>83 (23.1)	62 (8.5)<br>22 (6.1)	0.33
5. A living donor kidney transplant may strengthen the relationship between the donor and recipient.	<60 years<br>≥60 years	86 (11.6)<br>51 (11.4)	535 (72.1)<br>296 (65.9)	121 (16.3)<br>102 (22.7)	0.02
6. Approaching a potential donor who then says no will change the relationship between the two people.	<60 years<br>≥60 years	347 (46.8)<br>205 (45.9)	164 (22.1)<br>59 (13.2)	230 (31.0)<br>183 (40.9)	<0.001
7. Asking someone to donate makes the recipient seem selfish.	<60 years<br>≥60 years	393 (53.2)<br>176 (29.3)	210 (28.4)<br>129 (28.8)	136 (18.4)<br>143 (31.9)	<0.001
8. It is acceptable for a parent to receive a kidney from his/her child (over 18 years old).	<60 years<br>≥60 years	71 (9.6)<br>52 (11.6)	584 (78.7)<br>334 (74.4)	87 (11.7)<br>63 (14.0)	0.23
9. Decisions about donation should be made by the donor alone. The recipient should not ask for a kidney.	<60 years<br>≥60 years	223 (30.1)<br>69 (15.4)	420 (56.8)<br>328 (73.1)	97 (13.1)<br>52 (11.6)	<0.001
10. Since the donor operation is not risk free, someone who needs a kidney transplant should wait for a kidney from someone who has died.	<60 years<br>≥60 years	625 (84.4)<br>360 (80.2)	38 (5.1)<br>36 (8.0)	78 (10.5)<br>53 (11.8)	0.09

Table S3c. Difference in beliefs with participant education

Belief statement	Higher education level	Disagree (%)	Agree (%)	Don't know (%)	Chi^2 p-value
**1. It is morally acceptable to take a kidney from a healthy person.**	No university education<br>University education	34 (4.7)<br>21 (5.9)	637 (87.9)<br>322 (90.2)	54 (7.5)<br>14 (3.9)	0.06
**2. Donors often agree to donate due to feelings of guilt or family pressure.**	No university education<br>University education	388 (53.4)<br>181 (50.6)	137 (18.8)<br>72 (20.1)	202 (27.8)<br>105 (29.3)	0.68
**3. Donating a kidney is a rewarding experience for the live donors.**	No university education<br>University education	19 (2.6)<br>4 (1.1)	616 (84.4)<br>298 (83.2)	95 (13.0)<br>56 (15.6)	0.15
**4. Donating a kidney to someone requires an extremely close personal relationship.**	No university education<br>University education	492 (67.4)<br>254 (70.8)	176 (24.1)<br>83 (23.1)	62 (8.5)<br>22 (6.1)	0.33
**5. A living donor kidney transplant may strengthen the relationship between the donor and recipient.**	No university education<br>University education	94 (12.9)<br>26 (7.2)	504 (69.0)<br>276 (76.9)	132 (18.1)<br>57 (15.9)	0.008
**6. Approaching a potential donor who then says no will change the relationship between the two people.**	No university education<br>University education	358 (49.2)<br>151 (42.1)	122 (16.8)<br>82 (22.8)	248 (34.1)<br>126 (35.1)	0.03
**7. Asking someone to donate makes the recipient seem selfish.**	No university education<br>University education	332 (45.7)<br>188 (52.4)	204 (28.1)<br>105 (29.3)	190 (26.2)<br>66 (18.4)	0.01
**8. It is acceptable for a parent to receive a kidney from his/her child (over 18 years old).**	No university education<br>University education	75 (10.3)<br>38 (10.6)	568 (77.8)<br>282 (78.6)	87 (11.9)<br>39 (10.9)	0.87
**9. Decisions about donation should be made by the donor alone. The recipient should not ask for a kidney.**	No university education<br>University education	166 (22.7)<br>103 (28.9)	482 (66.0)<br>208 (58.3)	82 (11.2)<br>46 (12.9)	0.04
**10. Since the donor operation is not risk free, someone who needs a kidney transplant should wait for a kidney from someone who has died.**	No university education<br>University education	604 (82.9)<br>306 (85.2)	51 (7.0)<br>19 (5.3)	74 (10.2)<br>34 (9.5)	0.51

Table S3d. Difference in beliefs with participant ethnicity

Belief statement	Ethnicity	Disagree (%)	Agree (%)	Don't know (%)	Chi^2 p-value
**1. It is morally acceptable to take a kidney from a healthy person.**	White<br>BAME[a]	54 (5.4)<br>11 (6.6)	900 (89.1)<br>134 (80.7)	56 (5.5)<br>21 (12.7)	0.002
**2. Donors often agree to donate due to feelings of guilt or family pressure.**	White<br>BAME	535 (52.9)<br>83 (49.7)	192 (10.0)<br>31 (18.6)	285 (28.2)<br>53 (31.7)	0.63
**3. Donating a kidney is a rewarding experience for the live donors.**	White<br>BAME	22 (2.2)<br>4 (2.4)	855 (84.2)<br>129 (77.3)	138 (13.6)<br>34 (20.4)	0.07
**4. Donating a kidney to someone requires an extremely close personal relationship.**	White<br>BAME	697 (68.7)<br>104 (61.9)	244 (24.0)<br>43 (25.6)	74 (7.3)<br>21 (12.5)	0.05
**5. A living donor kidney transplant may strengthen the relationship between the donor and recipient.**	White<br>BAME	114 (11.2)<br>24 (14.3)	715 (70.4)<br>109 (64.9)	186 (18.3)<br>35 (20.8)	0.32
**6. Approaching a potential donor who then says no will change the relationship between the two people.**	White<br>BAME	471 (46.5)<br>81 (48.2)	190 (18.8)<br>29 (17.3)	351 (34.7)<br>58 (34.5)	0.88
**7. Asking someone to donate makes the recipient seem selfish.**	White<br>BAME	483 (47.7)<br>79 (47.3)	292 (28.9)<br>45 (27.0)	237 (23.4)<br>43 (25.8)	0.77
**8. It is acceptable for a parent to receive a kidney from his/her child (over 18 years old).**	White<br>BAME	104 (10.3)<br>16 (9.5)	787 (77.5)<br>127 (75.6)	124 (12.2)<br>25 (14.9)	0.62
**9. Decisions about donation should be made by the donor alone. The recipient should not ask for a kidney.**	White<br>BAME	247 (24.4)<br>43 (25.6)	649 (64.1)<br>99 (58.9)	117 (11.6)<br>26 (15.5)	0.28
**10. Since the donor operation is not risk free, someone who needs a kidney transplant should wait for a kidney from someone who has died.**	White<br>BAME	864 (85.2)<br>116 (69.1)	57 (5.6)<br>16 (9.5)	93 (9.2)<br>36 (21.4)	<0.001

[a] BAME - Black, Asian and Minority Ethnic group

Table S3e. Difference in beliefs with participant religion

Belief statement	Religion	Disagree (%)	Agree (%)	Don't know (%)	Chi^2 p-value
**1. It is morally acceptable to take a kidney from a healthy person.**	No religion	18 (5.5)	291 (89.3)	17 (5.2)	0.01
	Christian	33 (4.7)	626 (88.7)	47 (6.7)	
	Other religion[a]	10 (8.9)	88 (77.9)	15 (13.3)	
**2. Donors often agree to donate due to feelings of guilt or family pressure.**	No religion	161 (49.1)	71 (21.7)	96 (29.3)	0.22
	Christian	389 (55.0)	123 (17.4)	195 (27.6)	
	Other religion	54 (47.8)	27 (23.9)	32 (28.3)	
**3. Donating a kidney is a rewarding experience for the live donors.**	No religion	3 (0.9)	264 (80.0)	63 (19.1)	<0.001
	Christian	19 (2.7)	612 (86.4)	77 (10.9)	
	Other religion	4 (3.5)	82 (72.6)	27 (23.9)	
**4. Donating a kidney to someone requires an extremely close personal relationship.**	No religion	232 (70.3)	73 (22.1)	25 (7.6)	0.08
	Christian	481 (67.8)	176 (24.8)	52 (7.3)	
	Other religion	67 (59.3)	30 (26.6)	16 (14.2)	
**5. A living donor kidney transplant may strengthen the relationship between the donor and recipient.**	No religion	36 (10.9)	231 (70.0)	63 (19.1)	0.82
	Christian	83 (11.7)	501 (70.7)	125 (17.6)	
	Other religion	13 (11.5)	75 (66.4)	25 (22.1)	
**6. Approaching a potential donor who then says no will change the relationship between the two people.**	No religion	139 (42.1)	83 (25.2)	108 (32.7)	0.008
	Christian	344 (48.7)	111 (15.7)	251 (35.6)	
	Other religion	49 (43.4)	24 (21.2)	40 (35.4)	
**7. Asking someone to donate makes the recipient seem selfish.**	No religion	153 (46.5)	108 (32.8)	68 (20.7)	0.30
	Christian	350 (49.5)	188 (26.6)	169 (23.9)	
	Other religion	51 (45.5)	35 (31.3)	26 (23.2)	
**8. It is acceptable for a parent to receive a kidney from his/her child (over 18 years old).**	No religion	37 (11.2)	261 (79.1)	32 (9.7)	0.31
	Christian	69 (9.7)	542 (76.5)	98 (13.8)	
	Other religion	12 (10.6)	83 (73.5)	18 (15.9)	
**9. Decisions about donation should be made by the donor alone. The recipient should not ask for a kidney.**	No religion	82 (25.0)	205 (62.5)	41 (12.5)	0.32
	Christian	165 (23.3)	460 (64.9)	84 (11.9)	
	Other religion	36 (31.9)	62 (54.9)	15 (13.3)	
**10. Since the donor operation is not risk free, someone who needs a kidney transplant should wait for a kidney from someone who has died.**	No religion	292 (88.8)	11 (3.3)	26 (7.9)	<0.001
	Christian	594 (83.8)	46 (6.5)	69 (9.7)	
	Other religion	73 (64.6)	13 (11.5)	27 (23.9)	

[a] Muslim/Hindu/Jewish/Sikh/Buddhist/Other - combined due to small numbers including single participant responders in some groups risking identification.

Table S4. Comparison with participants in ATTOM study

ATTOM study - 72% of invited participants in final analysis

Bailey et al study - 40% of invited participants in final analysis

	**ATTOM participants**<br>**Likelihood of LDKT over DDKT**<br>**Unadjusted OR (p-value)**	**Participants in Bailey et al study**<br>**Likelihood of LDKT over DDKT**<br>**Unadjusted OR (p-value)**
**No qualifications**	Reference	Reference
**University**	0.39 (p <0.001)	0.46 (p=0.02)
**School level education**	Reference	Reference
**University education**	0.73 (p=0.009)	0.72 (p=0.05)

	**ATTOM participants**<br>**Likelihood of LDKT over DDKT**<br>**Adjusted OR (p-value)**	**Participants in Bailey et al study**<br>**Likelihood of LDKT over DDKT**<br>**Adjusted OR (p-value)**
**No qualifications**	Reference	Reference
**University**	0.55 (p <0.001)	0.48 (p=0.03)
**School level education**	Reference	Reference
**University education**	0.76 (p=0.01)	0.69 (p=0.001)
